# Major complications after percutaneous biopsy of native or transplanted liver in pediatric patients: a nationwide inpatient database study in Japan

**DOI:** 10.1186/s12876-022-02476-7

**Published:** 2022-08-24

**Authors:** Kayo Ikeda Kurakawa, Akira Okada, Kazuhiko Bessho, Taisuke Jo, Sachiko Ono, Nobuaki Michihata, Ryosuke Kumazawa, Hiroki Matsui, Kiyohide Fushimi, Satoko Yamaguchi, Toshimasa Yamauchi, Masaomi Nangaku, Takashi Kadowaki, Hideo Yasunaga

**Affiliations:** 1 Department of Prevention of Diabetes and Lifestyle-Related Diseases, Graduate School of Medicine, The University of Tokyo, Tokyo, Japan; 2 Present Address: Department of Pediatrics, National Rehabilitation Center for Persons With Disabilities, Saitama, Japan; 3 Department of Pediatrics, Graduate School of Medicine, Osaka University, Osaka, Japan; 4 Department of Health Services Research, Graduate School of Medicine, The University of Tokyo, Tokyo, Japan; 5 Department of Eat-Loss Medicine, Graduate School of Medicine, The University of Tokyo, Tokyo, Japan; 6 Department of Clinical Epidemiology and Health Economics, School of Public Health, The University of Tokyo, Tokyo, Japan; 7 Department of Health Policy and Informatics, Tokyo Medical and Dental University Graduate School of Medicine, Tokyo, Japan; 8 Department of Diabetes and Metabolism, Graduate School of Medicine, The University of Tokyo, Tokyo, Japan; 9 Department of Nephrology and Endocrinology, Graduate School of Medicine, The University of Tokyo, Tokyo, Japan; 10 Toranomon Hospital, 2-2-2, Toranomon, Minato-ku, Tokyo, 105-8470 Japan

**Keywords:** Pediatric percutaneous liver biopsy, Major complications, Impatient database study, Transplanted liver, Native liver

## Abstract

**Aim:**

Although major complication rates following percutaneous liver biopsy (PLB) have been reported to be higher in children than in adults, scarce data are available regarding pediatric patients stratified by native and transplanted liver. We aimed to assess the factors associated with major complications after percutaneous biopsy of native or transplanted liver using a nationwide inpatient database.

**Methods:**

Using the Japanese Diagnosis Procedure Combination database, we retrospectively identified pediatric patients who underwent PLB between 2010 and 2018. We described major complication rates and analyzed factors associated with major complications following PLB, stratified by native and transplanted liver.

**Results:**

We identified 3584 pediatric PLBs among 1732 patients from 239 hospitals throughout Japan during the study period, including 1310 in the native liver and 2274 in the transplanted liver. Major complications following PLB were observed in 0.5% (n = 18) of the total cases; PLB in the transplanted liver had major complications less frequently than those in the native liver (0.2% vs. 1.0%, *p* = 0.002). The occurrence of major complications was associated with younger age, liver cancers, unscheduled admission, anemia or coagulation disorders in cases with native liver, while it was associated with younger age alone in cases with transplanted liver.

**Conclusions:**

The present study, using a nationwide database, found that major complications occurred more frequently in pediatric cases with native liver and identified several factors associated with its major complications.

## Introduction

Although noninvasive tools, such as transient elastography have been developed, percutaneous liver biopsy (PLB) remains the gold standard method for determining the etiology of liver dysfunction or disorder in pediatric patients with native and transplanted liver. Complication rates following PLB in pediatric populations are reportedly higher than those in adult populations [1–4], and serious complications, such as bleeding, organ perforation, and death have been observed even in children [5–8]. Therefore, risk stratification for PLB complications is particularly important in pediatric patients.

Previous studies have shown that major complications after PLB occur in 0–4.6% of pediatric patients [1, 2, 6, 8–10], and that younger age and hepatoblastoma or hepatocellular carcinoma were associated with complications following PLB in pediatric patients [6, 7]. However, these studies did not consider whether PLB had been performed on native or transplanted liver. Several small studies (n = 120 or 219) focused on major complications after PLB on transplanted liver in pediatric patients, with an occurrence of 0.91% to 1.7% [11, 12].

Residual liver function, which can affect bleeding events after PLB, may differ between transplanted and native liver. Furthermore, the indication and transplanted liver biopsy site differed from those of the native liver. Thus, the risk assessment for PLB should be stratified by native or transplanted liver.

Previous studies including 200–300 children showed that the rate of minor bleeding after PLB was lower in those with transplanted liver than in those with native liver. However, owing to the limited number of included patients, these studies failed to examine major complications [6, 7]. Thus, it remains unknown whether PLB in the transplanted liver is associated with lower rates of major complications than that in the native liver.

In the present study, using a nationwide inpatient database in Japan, we aimed to investigate the major complication rates in pediatric PLB stratified by native or transplanted liver. We also analyzed the factors associated with major complications following PLB.

## Methods

### Data source

We used the Diagnosis Procedure Combination database, a nationwide inpatient database in Japan [13]. Details of the database have been reported in a previous study [14]. The database contains inpatient administrative claims and discharge abstracts for more than 1200 hospitals, and covers all academic hospitals and over 90% of all tertiary-care emergency hospitals in Japan. The database includes the following information: age, sex, height, and body weight; diagnoses (primary admission-precipitating diagnosis, comorbidities present on admission, and in-hospital complications); medical procedures; medications and devices used; and discharge status (discharge home, discharge to another hospital, or in-hospital death).

All diagnoses were recorded using the International Classification of Diseases, 10th revision (ICD-10) codes and free-text data.

This study was approved by the Institutional Review Board of the Graduate School of Medicine of the University of Tokyo. All methods were carried out in accordance with relevant guidelines and regulations.

### Study population and eligibility criteria

We included all PLBs performed within five days of admission among pediatric patients (aged < 18 years) admitted between July 2010 and March 2018 in the database.

We excluded PLB in pregnant women or PLB on admission when the following procedures were performed: liver transplantation, any surgeries before PLB, or surgeries irrelevant to major complications (presence of any surgeries other than vascular embolization or ligation, repair for pneumothorax or hemothorax, or exploratory laparotomy or thoracotomy) within six days following PLB. We also excluded patients who underwent transfusion before PLB or those with missing information on body weight or height.

### Study variables and outcomes

We collected the following information: age, sex, body mass index (BMI), use of corticosteroid or immunosuppressant before PLB, pediatric score in complex chronic condition (CCC) classification system version 2 [15], comorbidity with anemia or coagulation disorders (ICD-10 codes: D46, D50-64, or D66-69) present on admission, presence of any cancer other than liver cancer, etiology of liver disease recorded in the database, liver transplant rejection (ICD-10 codes: T864, or T869), unscheduled admission, type of anesthesia used during PLB (with or without general anesthesia), hospital volume for pediatric PLB (average annual number of pediatric patients who underwent PLB), and history of previous PLB during the study period. Age was categorized into four groups: < 1, 1–5, 6–11, and 12–17 years old. The BMI was categorized using cutoffs of 18.5 kg/m^2^ and 25.0 kg/m^2^ in patients aged 17 years; otherwise, the BMI was categorized according to BMI standard deviation score (BMI-SDS). The BMI-SDS was calculated using BMI, sex, and age in months to assess the BMI in an age- and sex-dependent manner, as described in details previously [16]. The patients were classified as underweight (BMI-SDS < − 1.28), normal weight (BMI-SDS − 1.28 to 1.279), and overweight or obesity (BMI-SDS ≥ 1.28) [17, 18]. The CCCs were calculated based on the number of diseases among 12 disease categories to adjust for the severity of comorbidities on admission, as previously described [15, 19]. The disease categories used in CCCs are as follows: neurological and neuromuscular, cardiovascular, respiratory, renal and urologic, gastrointestinal, hematologic and immunologic, metabolic, other congenital and genetic defects, malignancies, premature and neonatal disorders, technology dependence (use of medical devices), and transplantation (ICD-10 codes: T86, failure and rejection of transplanted organs and tissues). The etiology of liver disease in patients with native liver included acute liver failure (ICD-10 codes: B150, B162, B190, K711, K720, or K729), liver cirrhosis (ICD-10 codes: B181, K703, K717, or K74), biliary atresia (ICD-10 codes: Q442 or Z090), inborn errors of metabolism (ICD-10 codes: E72-77, E80, E83, or B888), autoimmune hepatitis (ICD-10 codes: K754), and liver cancers (ICD-10 codes: C22). General anesthesia was defined as anesthesia with intubation. We categorized hospital volume into three categories based on the tertiles of hospitals. We defined patients with a transplanted liver using ICD-10 codes Z944, T864, or T869.

The primary outcome was major complications, defined as the occurrence of major bleeding or organ perforations necessitating transfusions or surgical interventions (vascular embolization or ligation, repair for pneumothorax or hemothorax, and exploratory laparotomy or thoracotomy) within six days of liver biopsy, as previously reported [3, 6, 20]. In-hospital death was observed in a very small number of patients undergoing major complications following PLB; however, we did not describe who died during hospitalization for protection of personal information.

### Statistical analysis

We summarized the clinical characteristics of cases with native and transplanted liver and evaluated factors associated with major complications following PLB in each population. We also described the occurrence of major complications within six days after PLB among the total cases. Continuous variables were reported as median (interquartile range) and categorical variables were reported as numbers (percentage). To compare the proportions of the two groups, we used the Fisher’s exact test when the expected number in a cell was < 10; otherwise, the chi-squared test was used. To consider the effect of the difference in age or sex distributions on the major complication rates, we performed analyses for major complication rates stratified by sex or age in cases with native liver or transplanted liver. For the stratified analyses, age was divided into two groups by the median of the total population. The threshold for significance was set at *p* < 0.05. All statistical analyses were performed using Stata software version 17 (Stata Corp., College Station, TX, USA).

## Results

**Fig. 1:**
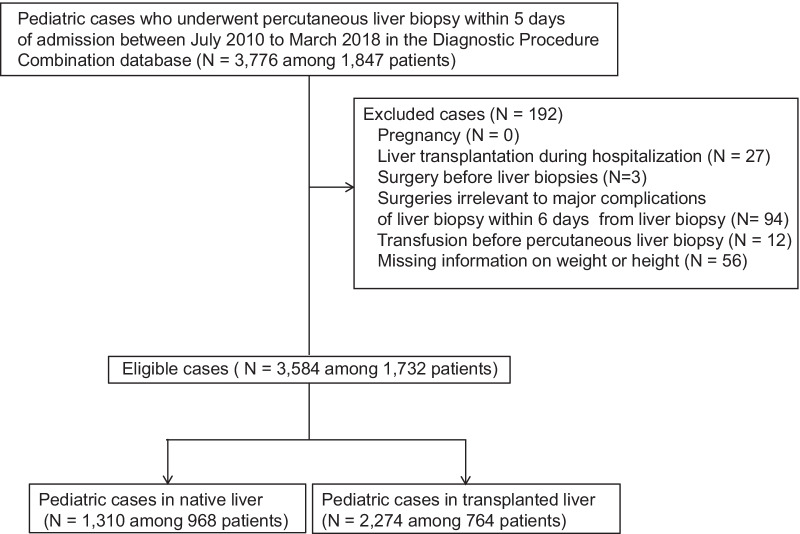
Flow chart showing the selection of cases. Abbreviation: BMI-SDS, body mass index standard deviation score

We identified 3776 PLBs performed in 1847 patients during the study period, and 192 observations were excluded based on the exclusion criteria (Fig. 1). Thus, 3584 PLBs for 1732 patients from 239 hospitals were included in the study.

Table 1 shows the clinical characteristics of 1310 (36.6%) PLBs among 968 patients with native liver or 2274 (63.4%) PLBs among 764 patients with transplanted liver. Among the cases with native liver, biliary atresia was the most common liver disease on admission (n = 239, 18.2%). In comparison to PLB in native liver, cases with transplanted liver were more likely to be females (57.5% vs. 44.0%, *p* < 0.001) and younger. The median age of the total cases was 10 years (inter-quartile range 5–14 years). The BMI was lower in the PLB of the transplanted liver. We found a higher proportion of corticosteroid use (12.7% vs. 3.2%, *p* < 0.001) and immunosuppressant use (33.4% vs. 4.3%, *p* < 0.001) among PLB in the transplanted liver. Regarding comorbidities, we did not find a significant difference in the proportion of those with anemia or coagulation diseases (5.0% vs. 6.2%, *p* = 0.11) or in the proportion of those with cancers other than liver cancer (0.9% vs. 0.6%, *p* = 0.31), whereas the CCC score (17.1% vs. 14.0%, *p* = 0.012) was higher in PLB among the native liver patients. The proportion of patients undergoing an unscheduled admission did not differ significantly between the groups (6.5% vs. 8.0%, *p* = 0.088). Cases with a transplanted liver were more likely to have a history of previous PLB (65.7% vs. 23.6%, *p* < 0.001). Major complications were observed in 0.5% (n = 18) of the total PLB population, and were significantly lower in the transplanted liver (0.2% vs. 1.0%, *p* = 0.002).

**Table 1:** Clinical characteristics of pediatric cases with native liver and transplanted liver who underwent percutaneous liver biopsies

Characteristics	Category	Native liver (N = 1310)	Transplanted liver (N = 2274)	Total (N = 3584)	*p*-value
Sex	Male	734 (56.0)	967 (42.5)	1701 (47.5)	< 0.001
Age (years)		10 (5–15)	9 (5–13)	10 (5–14)	< 0.001
Age (years)	< 1	48 (3.7)	17 (0.7)	65 (1.8)	< 0.001
	1–5	320 (24.4)	650 (28.6)	970 (27.1)	
	6–11	360 (27.5)	797 (35.0)	1157 (32.3)	
	12–17	582 (44.4)	810 (35.6)	1392 (38.8)	
BMI or BMI-SDS	Underweight	140 (10.7)	275 (12.1)	415 (11.6)	< 0.001
	Normal	929 (70.9)	1825 (80.3)	2754 (76.8)	
	Overweight or obesity	241 (18.4)	174 (7.7)	415 (11.6)	
CCC score	≤ 1	1086 (82.9)	1956 (86.0)	3042 (84.9)	0.012
	≥ 2	224 (17.1)	318 (14.0)	542 (15.1)	
Comorbidities on admission	Anemia or coagulation diseases	65 (5.0)	142 (6.2)	207 (5.8)	0.11
	Cancers other than liver cancer	12 (0.9)	14 (0.6)	26 (0.7)	0.31
	Acute liver failure	53 (4.0)	N/A	N/A	N/A
	Liver cirrhosis	155 (11.8)	N/A	N/A	N/A
	Biliary atresia	239 (18.2)	N/A	N/A	N/A
	Inborn errors of metabolism	154 (11.8)	N/A	N/A	N/A
	Autoimmune hepatitis	87 (6.6)	N/A	N/A	N/A
	Liver cancer	17 (1.3)	N/A	N/A	N/A
	Transplant rejection	N/A	659 (29.0)	N/A	N/A
Unscheduled admission		85 (6.5)	183 (8.0)	268 (7.5)	0.088
Corticosteroids use		42 (3.2)	289 (12.7)	331 (9.2)	< 0.001
Immunosuppressant use		56 (4.3)	760 (33.4)	816 (22.8)	< 0.001
Type of anesthesia	General anesthesia	447 (34.1)	425 (18.7)	872 (24.3)	< 0.001
	Without general anesthesia	860 (65.6)	1848 (81.3)	2708 (75.6)	
	Unknown	3 (0.2)	1 (0.0)	4 (0.1)	
Previous liver biopsies history		309 (23.6)	1495 (65.7)	1804 (50.3)	< 0.001
Hospital volume per year (cases)	1–17	830 (63.4)	349 (15.3)	1179 (32.9)	< 0.001
	18–54	339 (25.9)	771 (33.9)	1110 (31.0)	
	≥ 55	141 (10.8)	1154 (50.7)	1295 (36.1)	
Major complications		13 (1.0)	5 (0.2)	18 (0.5)	0.002

Age is presented as median (interquartile range). All the other data are shown as n (%)

*BMI* body mass index, *BMI-SDS* body mass index standard deviation score, *CCC score* pediatric score of complex chronic conditions classification system, *N/A* not applicable

Table 2 focuses on cases with native liver, showing the characteristics stratified by major complications following PLB during hospitalization. We identified 13 pediatric cases of major PLB complications. Major complications were frequently observed in younger patients, in cases with anemia or coagulation diseases on admission, in cases with liver cancer, or in cases undergoing unscheduled admission. No association was observed between acute liver failure or liver cirrhosis and major complications.

**Table 2:** Characteristics of pediatric cases with native liver who underwent percutaneous liver biopsy stratified by major complications during hospitalization

Characteristics	Category	Cases without major complication (N = 1297)	Cases with major complication (N = 13)	Total (N = 1310)	*p*-value
Sex	Male	728 (56.1)	6 (46.2)	734 (56.0)	0.58
Age (years)		10 (5–15)	3 (0–10)	10 (5–15)	0.006
Age (years)	< 1	44 (3.4)	4 (30.8)	48 (3.7)	< 0.001
	1–5	316 (24.4)	4 (30.8)	320 (24.4)	
	6–11	357 (27.5)	3 (23.1)	360 (27.5)	
	12–17	580 (44.7)	2 (15.4)	582 (44.4)	
BMI or BMI-SDS	Underweight	137 (10.6)	3 (23.1)	140 (10.7)	0.24
	Normal	920 (70.9)	9 (69.2)	929 (70.9)	
	Overweight or obesity	240 (18.5)	1 (7.7)	241 (18.4)	
CCC score	≤ 1	1075 (82.9)	11 (84.6)	1086 (82.9)	≥ 0.99
	≥ 2	222 (17.1)	2 (15.4)	224 (17.1)	
Comorbidities on admission	Anemia or coagulation diseases	61 (4.7)	4 (30.8)	65 (5.0)	0.003
	Cancers other than liver cancer	12 (0.9)	0 (0.0)	12 (0.9)	≥ 0.99
	Liver cancer	11 (0.8)	6 (46.2)	17 (1.3)	< 0.001
	Acute liver failure	52 (4.0)	1 (7.7)	53 (4.0)	0.42
	Liver cirrhosis	154 (11.9)	1 (7.7)	155 (11.8)	≥ 0.99
	Biliary atresia	239 (18.4)	0 (0.0)	239 (18.2)	0.14
	Inborn errors of metabolism	151 (11.6)	3 (23.1)	154 (11.8)	0.19
	Autoimmune hepatitis	86 (6.6)	1 (7.7)	87 (6.6)	0.59
Unscheduled admission		82 (6.3)	3 (23.1)	85 (6.5)	0.047
Corticosteroid use		42 (3.2)	0 (0.0)	42 (3.2)	≥ 0.99
Immunosuppressant use		56 (4.3)	0 (0.0)	56 (4.3)	≥ 0.99
Previous liver biopsies history		308 (23.7)	1 (7.7)	309 (23.6)	0.32
Hospital volume per year (cases)	1–17	823 (63.5)	7 (53.8)	830 (63.4)	0.33
	18–54	336 (25.9)	3 (23.1)	339 (25.9)	
	≥ 55	138 (10.6)	3 (23.1)	141 (10.8)	

Age is presented as median (interquartile range). All the other data are shown as n (%)

*BMI* body mass index, *BMI-SDS* body mass index standard deviation score, *CCC score* pediatric score of complex chronic conditions classification system

The characteristics of PLB in the transplanted liver stratified by major complications during hospitalization are described in Table 3, and we identified five cases with major complications. The major complication rate was higher in younger cases. Although not statistically significant, corticosteroid use before PLB was associated with major complications (*p* = 0.12). Table 4 shows the major complication rates stratified by sex or age in cases with native liver or transplanted liver. Major complication rates were lower in cases with transplanted liver than that with native livers, although we did not find statistical significance in all sub-group analyses.

**Table 3:** Characteristics of pediatric cases with transplanted liver who underwent percutaneous liver biopsy stratified by major complications during hospitalization

Characteristics	Category	Cases without major complication (N = 2269)	Cases with major complication (N = 5)	Total (N = 2274)	*p*-value
Sex	Male	965 (42.5)	2 (40.0)	967 (42.5)	≥ 0.99
Age (years)		9 (5–13)	1 (1–1)	9 (5–13)	0.017
Age (years)	< 1	16 (0.7)	1 (20.0)	17 (0.7)	0.004
	1–5	647 (28.5)	3 (60.0)	650 (28.6)	
	6–11	797 (35.1)	0 (0.0)	797 (35.0)	
	12–17	809 (35.7)	1 (20.0)	810 (35.6)	
BMI or BMI-SDS	Underweight	275 (12.1)	0 (0.0)	275 (12.1)	0.42
	Normal	1821 (80.3)	4 (80.0)	1825 (80.3)	
	Overweight or obesity	173 (7.6)	1 (20.0)	174 (7.7)	
CCC score	≤ 1	1952 (86.0)	4 (80.0)	1956 (86.0)	0.53
	≥ 2	317 (14.0)	1 (20.0)	318 (14.0)	
Comorbidities on admission	Anemia or coagulation diseases	142 (6.3)	0 (0.0)	142 (6.2)	≥ 0.99
	Cancers other than liver cancer	14 (0.6)	0 (0.0)	14 (0.6)	≥ 0.99
	Transplant rejection	658 (29.0)	1 (20.0)	659 (29.0)	≥ 0.99
Unscheduled admission		182 (8.0)	1 (20.0)	183 (8.0)	0.34
Corticosteroid use		287 (12.6)	2 (40.0)	289 (12.7)	0.12
Immunosuppressant use		758 (33.4)	2 (40.0)	760 (33.4)	≥ 0.99
Previous liver biopsies history		1492 (65.8)	3 (60.0)	1495 (65.7)	≥ 0.99
Hospital volume per year (cases)	1–17	348 (15.3)	1 (20.0)	349 (15.3)	0.71
	18–54	770 (33.9)	1 (20.0)	771 (33.9)	
	≥ 55	1151 (50.7)	3 (60.0)	1154 (50.7)	

Age is presented as median (interquartile range). All the other data are shown as n (%)

*BMI* body mass index, *BMI-SDS* body mass index standard deviation score, *CCC score* pediatric score of complex chronic conditions classification system

**Table 4:** Major complication rates following pediatric percutaneous liver biopsy on native or transplanted liver in sex or age-stratified analysis

Characteristics	Category	Cases with major complication in native liver (N = 13)	Cases with major complication in transplanted liver (N = 5)	*p*-value
Sex	Male (N = 1701)	6/734 (0.8)	2/967 (0.2)	0.083
	Female (N = 1883)	7/576 (1.2)	3/1307 (0.2)	0.012
Age (years)	< 10 (N = 1764)	9/592 (1.5)	4/1172 (0.3)	0.014
	≥ 10 (N = 1820)	4/718 (0.6)	1/1102 (0.1)	0.083

All data are shown as number of patients reaching events/total number of patients analyzed in subgroups (%)

**Fig. 2:**
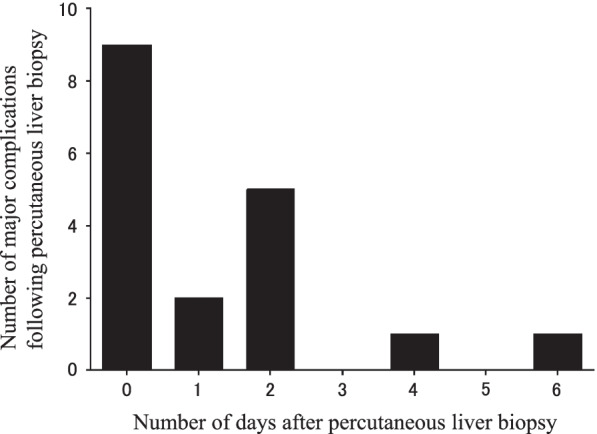
Histogram that shows when major complications occurred following PLB among the total cases. Abbreviation: PLB, percutaneous liver biopsy

Figure 2 summarizes the major complications that occurred after PLB in all cases. Almost 90% of major complications occurred ≤ 2 days after the PLB. All the surgical interventions were performed on the day of or day after PLB. Vascular embolization, vascular ligation, exploratory laparotomy, and exploratory thoracotomy were performed in three, one, two, and one case, respectively. On the other hand, we found two cases who received transfusion four and six days after the PLB.

## Discussion

Using a national inpatient database, including 3584 pediatric PLB on native and transplanted liver, we evaluated the major complication rates and factors associated with major complications following PLB in each population. The major complication rates were lower in cases with transplanted liver than in those with native liver. Several factors were associated with major complications in the native liver. However, only a younger age was identified in cases with transplanted liver.

The overall proportion of major complications among the total cases with both types of liver (0.5%) was similar to that in previous reports on pediatric populations [5, 21].

In Japan, nearly all pediatric patients are admitted to a hospital for liver biopsy. Therefore, we believe that our results reflect the overall results of pediatric liver biopsies in Japan, observing real-world evidence on major complication rates. We observed a lower rate of major complications among those with transplanted liver than among those with native liver. Similar patterns were observed even on sex- or age- stratified analyses. Therefore, the differences in sex or age distribution between cases with native liver and transplanted liver did not confound the study results. Although the reasons for lower complication rates in cases with transplanted liver may be uncertain, relatively preserved liver function or the absence of liver cancer in the transplanted liver may contribute to the lower rate of major complications. Additionally, PLB is usually performed with a substernal approach in pediatric patients with transplanted liver [11, 12, 22], and compression hemostasis following PLB may be easier.

Major complications following PLB in the cases with native liver were associated with younger age, anemia or coagulation diseases, liver cancer, and unscheduled admission. Liver cirrhosis was not associated with major complications. To our knowledge, no previous study has analyzed factors associated with major complications in pediatric patients with native or transplanted liver separately. Even in adult patients, there was only one study focusing on bleeding complications following PLB in patients with native liver, which reported that liver cancer and advanced liver cirrhosis were possible risk factors for bleeding complications [20].

A previous study using 451 pediatric patients with transplanted liver under stable conditions (i.e., without high doses of corticosteroids) reported no major bleeding complication [23]. Another previous study identified younger age as a possible risk factor in pediatric patients with native and transplanted liver [7]. In the present study, five cases with major complications were observed out of 2274 PLB in the transplanted iver, and only younger age was associated with major complications. We included patients with a high risk for bleeding, such as those treated with corticosteroids [20], which may have contributed to the five cases. This rare occurrence may have contributed to the failure to identify factors associated with major complications in patients with transplanted liver.

The strength of the present study is that we used a national database to evaluate major complication occurrences and factors associated with major complications in pediatric cases with native and transplanted liver.

This study had several limitations. First, the database did not contain clinical information on laboratory data for hemoglobin level, bleeding time, platelet count, or coagulation ability. However, we considered disease names, such as anemia or coagulation disorders and adopted an objective outcome of transfusion instead of a decline in the hemoglobin level. Second, information about the number of needle passes on same day as the PLB was unavailable, which was associated with major complications in a previous study [3]. Third, because of our small number of cases with major complications out of 3584 PLB cases in total, we could not evaluate the risk factors for major complications using multivariable analysis.

In conclusion, the present study, using a Japanese nationwide inpatient database, showed that pediatric PLB was a safe procedure, especially in patients with transplanted liver. We evaluated factors associated with major complications among pediatric cases with native liver and transplanted liver separately. Younger age, comorbidities with liver cancer, anemia or coagulation disorders, and unscheduled admission were associated with major complications in cases with native liver. In cases with transplanted liver, only a younger age was associated with major complications following PLB.

## Data Availability

The datasets analyzed during the current study are not publicly available because of contracts with hospitals that provide data to the database but are available from the corresponding author on reasonable request.
